# MtrA of the sodium ion pumping methyltransferase binds cobalamin in a unique mode

**DOI:** 10.1038/srep28226

**Published:** 2016-06-21

**Authors:** Tristan Wagner, Ulrich Ermler, Seigo Shima

**Affiliations:** 1 Max Planck Institute for Terrestrial Microbiology, Karl-von-Frisch-Straße 10, 35043 Marburg, Germany; 2 Max Planck Institute of Biophysics, Max-von-Laue-Straße 3, 60438 Frankfurt am Main, Germany; 3 PRESTO, Japan Science and Technology Agency (JST), 4-1-8 Honcho Kawaguchi, 332-0012 Saitama, Japan

## Abstract

In the three domains of life, vitamin B_12_ (cobalamin) is primarily used in methyltransferase and isomerase reactions. The methyltransferase complex MtrA–H of methanogenic archaea has a key function in energy conservation by catalysing the methyl transfer from methyl-tetrahydromethanopterin to coenzyme M and its coupling with sodium-ion translocation. The cobalamin-binding subunit MtrA is not homologous to any known B_12_-binding proteins and is proposed as the motor of the sodium-ion pump. Here, we present crystal structures of the soluble domain of the membrane-associated MtrA from *Methanocaldococcus jannaschii* and the cytoplasmic MtrA homologue/cobalamin complex from *Methanothermus fervidus*. The MtrA fold corresponds to the Rossmann-type α/β fold, which is also found in many cobalamin-containing proteins. Surprisingly, the cobalamin-binding site of MtrA differed greatly from all the other cobalamin-binding sites. Nevertheless, the hydrogen-bond linkage at the lower axial-ligand site of cobalt was equivalently constructed to that found in other methyltransferases and mutases. A distinct polypeptide segment fixed through the hydrogen-bond linkage in the relaxed Co(III) state might be involved in propagating the energy released upon corrinoid demethylation to the sodium-translocation site by a conformational change.

In the methanogenic pathway from H_2_ and CO_2_, the membrane-spanning methyl-tetrahydromethanopterin (CH_3_-H_4_MPT):coenzyme M (CoM-SH) methyltransferase complex (MtrA–H) catalyses the exergonic methyl transfer from CH_3_-H_4_MPT to CoM-SH (ΔG°′ = −30 kJ/mol) coupled with endergonic sodium-ion gradient formation^1^. In methanogens lacking cytochromes, the MtrA–H reaction represents the only chemiosmotic process^1^,^2^,^3^. The generated electrochemical membrane potential is used for ATP biosynthesis from ADP and inorganic phosphate by A_1_A_O_ ATP synthase^4^.

The molecular organization of the MtrA−H complex and its catalysed reactions are outlined in Fig. 1 (see also Supplementary Figure S1). Primary structure analysis predicts MtrC, MtrD and MtrE as integral membrane proteins with at least six transmembrane helices, MtrA, MtrB, MtrF and MtrG as peripheral proteins with one transmembrane helix anchor, and MtrH as a peripheral protein without a membrane anchor^1^,^5^,^6^. The only prosthetic group required for catalysis is vitamin B_12_ (cobalamin) bound to MtrA. According to a previous electron paramagnetic resonance (EPR) spectroscopy and site-directed mutagenesis analyses^7^,^8^,^9^,^10^,^11^, cobalt of the corrinoid in the inactive Co(II) state is axially coordinated by a histidine (His84 of MtrA from *Methanothermobacter marburgensis*) imidazolium group (base-off/His-on configuration) rather than by the dimethylbenzimidazole base as a fifth ligand^9^,^10^,^11^.

The MtrA–H reaction starts with a methyl group transfer from methyl-H_4_MPT bound to MtrH to the upper ligation site of cob(I)alamin (ΔG°′ = approximately −15 kJ/mol)^1^,^6^. Subsequently, the methyl group of the generated methyl-cob(III)alamin is transferred to CoM-SH in the presence of sodium ions (ΔG°′ = approximately −15 kJ/mol)^1^,^8^. This finding points towards the participation of the demethylation reaction in sodium-ion translocation^12^, which is probably localized on MtrE as it contains a characteristic aspartate in the transmembrane helix^1^,^5^. Based on the intensively studied coordination and redox chemistry of cobalamins, the histidine ligand is not coordinated to cobalt in the reduced non-methylated cob(I)alamin form (base-off/His-off), which is in contrast to the methylated cob(III)alamin form (base-off/His-on)^1^,^8^,^13^. Therefore, it has been proposed that the dissociation of the His ligand during formation of cob(I)alamin from methyl-cob(III)alamin induces conformational rearrangements of parts of MtrA^1^. From there, the energy is transmitted to MtrE, thereby causing sodium ion translocation (Fig. 1 and Supplementary Fig. S2)^12^.

Enzymes carrying a prosthetic group derived from vitamin B_12_ (referred to as B_12_ enzymes) can be grouped into those containing a Rossmann fold-like open α/β domain^14^,^15^ (termed Rossmann-type domain) such as methionine synthase^16^, methanol cobalamin methyltransferases^17^, iron-sulphur corrinoid methyltransferases^18^,^19^, methyl-malonyl-CoA mutases^20^ and glutamate mutases^21^, and those with other folds, such as reductive dehalogenases^22^ and glycerol and diol dehydratases^23^,^24^. Vitamin B_12_-dependent trafficking and membrane transport proteins were not taken into account. Methyltransferases and mutases contain the consensus sequence DXHXXG-41−42-SXL-26−28-GG for binding cobalamin^1^,^16^,^17^,^25^; although iron-sulphur cobalamin methyltransferases are the exception^18^,^19^. MtrA has no overall sequence similarity to any B_12_ enzyme and lacks the common fingerprint motif.

Genome analysis has revealed an extra copy or copies of the *mtrA* gene in many methanogenic archaea (*e.g., M. marburgensis*, *Methanobacterium formicicum*, *Methanobrevibacter ruminantium*, *Methanothermus fervidus* and *Methanosarcina barkeri*). These cytoplasmic MtrA homologues consist only of a soluble cobalamin-binding domain of 170–180 amino acids, which is in contrast to the approximately 250 amino acids of membrane-associated MtrA. Truncated MtrA and MtrA homologues of methanogens have a sequence identity of 60–97%. MtrA homologues have also been identified in the genomes of some bacteria and non-methanogenic archaea. Despite their low sequence identity compared to membrane-associated MtrA (~40%), the key residue His84 is mostly conserved.

Membrane-associated MtrA devoid of the C-terminal transmembrane helix was over-produced in *Escherichia coli* 20 years ago in a soluble but cobalamin-free form, and the holoenzyme was reconstituted by unfolding and refolding in the presence of cobalamin^11^. However, cobalamin appears to bind less tightly to isolated MtrA than to MtrA in the MtrA–H complex. Here, we report on the X-ray structures of the B_12_-binding domain of membrane-associated MtrA from *Methanocaldococcus jannaschii* and of the MtrA homologue from *M. fervidus* in complex with cobalamin. Analyses of these structures reveal a cobalamin-binding site that differs from other B_12_-binding proteins in an unexpected manner and also provides initial hints of how the energy of cobalt demethylation is transformed into conformational energy used for the ultimate sodium-ion translocation process.

## Results and discussion

### Experimental basis

To structurally characterize cobalamin-binding MtrA, we overproduce the soluble domain of MtrA of *M. jannaschii*, *M. marburgensis*, *M. evestigatum* and *M. kandleri* and the cytoplasmic MtrA homologues from *M. fervidus* and *M. evestigatum*. The use of the cytoplasmic MtrA homologues as models for MtrA in order to increase the crystallization space is justified because of the high sequence identity, e.g. 97% between membrane-associated MtrA and the MtrA homologue of *M. fervidus*. Because of the stability of bound B_12_, we used the MtrA homologue of *M. fervidus* and the genetically truncated MtrA of *M. jannaschii* (see Materials and methods).

The cytoplasmic MtrA homologue from *M. fervidus* was produced by heterologous expression as the cobalamin-free apoenzyme. The holoenzyme was subsequently reconstituted after unfolding and refolding by dilution with a buffer containing either methylcobalamin or hydroxocobalamin. Both refolded MtrA solutions were pink and showed characteristic UV/Vis spectra of cob(III)alamin (Supplementary Fig. S3). After 1 day of incubation, the reconstituted MtrA homologue‒hydroxocobalamin complex solution turned orange; the UV-Vis spectrum revealed the cob(II)alamin state (Supplementary Fig. S3)^26^. The tight binding of cobalamin to the MtrA homologue was verified by gel filtration and SDS-PAGE of unheated samples (Supplementary Fig. S4)^11^. Crystals of the MtrA homologue containing hydroxocobalamin grew after one year, and a unique crystal diffracted to 3.0 Å resolution. Phases were determined by using the single-wavelength anomalous dispersion method with cobalt as the anomalous scatterer. The structure revealed three cytoplasmic MtrA molecules in an asymmetric unit arranged as a homotrimer with a three-fold symmetry (Fig. 2). The trimer was mainly linked together by a covalent bond between the cobalt of cobalamin of one molecule and His35 of its neighbour. Cytoplasmic MtrA in complex with methyl-cobalamin did not crystallize under these conditions; the methyl group bound to cobalamin may prevent the coordination between cobalt and His35 of the neighbouring molecule. The occupancy of the upper axial ligation site by a histidine is presumably not physiologically relevant because the enzyme needs to switch from Co(I) to CH_3_-Co(III) during the reaction^1^. The absence of a significant contact area between the subunits is in line with this assumption (Fig. 2b).

The soluble domain of membrane-associated MtrA from *M. jannaschii* lacking the transmembrane helix was heterologously expressed in *E. coli* (Supplementary Fig. S5). We were again able to incorporate cobalamin into the apoenzyme by unfolding and refolding the protein, as verified by UV/Vis spectroscopy. Crystallization failed, possibly because the flexible part of the juxtamembrane segment (amino acids 161–220, Supplementary Fig. S5) between the transmembrane helix and the B_12_ domain disturbed the crystallization process. We therefore partially digested reconstituted MtrA with trypsin to remove this region. Unfortunately, the cobalamin content decreased to less than 50%, which suggested a role of the juxtamembrane segment in stabilizing the prosthetic group. The proteolysis product of MtrA yielded colourless cobalamin-free crystals that diffracted to 1.85 Å resolution (Supplementary Table 1). The structure was solved by molecular replacement using the model of cytoplasmic MtrA homologue as a search template. A malate molecule from the crystallization solution was bound to the binding site of the phosphate group of cobalamin of the MtrA homologue (Supplementary Fig. S6). The overall structures of the two MtrA variants were almost identical (Fig. 3), which was reflected by a root mean square deviation (rmsd) of 0.37 Å for a 127 C_α_ backbone and a sequence identity of 65%.

### Overall architecture of MtrA

The two MtrA variants studied here consist of a single domain with a Rossmann-like open α/β fold (Fig. 4a) that, except for dioldehydratases^23^,^24^, ribonucleotide reductases^27^ and reductive dehalogenases^22^, is characteristic of B_12_ enzymes (such as the cobalamin-containing fragment of methionine synthase from *E. coli*, MetH, shown in Fig. 4b). Although they share a common core topology motif β2α1β1α2β3α3β4α4 (Fig. 4), the β-sheet–α-helix arrangements, strand twisting and secondary structure linkers drastically vary between MtrA and other Rossmann-type B_12_ domains. In fact, MtrA is structurally more related to the functionally distinct pyruvate dehydrogenase (PDB code: 1ik6) or succinyl-CoA synthetase (PDB code: 1euc) domains than to any B_12_ enzyme (Supplementary Fig. S7). The Z-scores^28^ of the superimposed structures of MtrA and pyruvate dehydrogenase (PDB code: 1ik6) and succinyl-CoA synthase (PDB code: 1euc) are 8.4 and 8.1; the Z-scores of MtrA and the most related B_12_ domains of methionine synthase (1bmt) and methanol cobalamin methyltransferase (2i2x) are 4.5 and 5.5, respectively. The B_12_-binding protein, which has the highest Z-score (5.6) to MtrA, is monomethylamine corrinoid protein from *M. barkeri* (3ezx).

In MtrA, the core motif is extended by an N-terminally fused segment that is partly associated as an antiparallel β-strand (β0) to the central β-sheet and an expanded insertion region between helix α3 and strand β4 consisting of a unique meander-like segment and an α-helix (α3′). β0 and the unique meander-like segment are involved in cobalamin binding (Fig. 4a). The B_12_ domains of the corrinoid iron-sulphur proteins also contain an extra N-terminal antiparallel strand, whereas those of methanol cobalamin methyltransferase, methionine synthetase (MetH) and mutases are alternatively extended at the C-terminal end of the shared core by one strand (β5) and one helix (α5) (Fig. 4b).

The C-terminal stretch (amino acids 156–170) of truncated MtrA from *M. jannaschii*, which is classified as part of the juxtamembrane segment, turns back from helix α4 towards the C-terminal side of the central β-sheet (Fig. 3). This chain trace does not appear to be due to crystal lattice effects because several salt bridges and hydrophobic contacts between this stretch and the core domain were formed. The remaining 40–50 residues of the juxtamembrane segment appear to be flexible, at least in the truncated form because of the accessibility of this segment for proteolytic cleavage. A flexible connection of the B_12_ domain to the transmembrane helix is functionally useful for juggling between the methyl donor and acceptor in analogy to methionine synthetase^25^, corrinoid iron-sulphur protein methyltransferase^18^ and methanol-corrinoid methyltransferase^17^, although parts of the juxtamembrane segment might be attached to other subunits of the MtrA–H complex.

### The cobalamin binding mode

The cobalamin bound to the cytoplasmic MtrA homologue was positioned at the C-terminal region of the central β-sheet in a base-off/His-on configuration, which confirms previous EPR and mutational analyses^11^. Cobalamin was bound into a largely preformed binding site inferred from the highly similar structures of the cobalamin-loaded and -unloaded MtrA variants. The corrinoid ring sat exposed on the top of the surface loops following strands β2 and β3 and helix α3 (Fig. 4a), and it was mainly anchored to the polypeptide by the covalent bond between the corrinoid cobalt and His84-ND1 (at a distance of 2.7 Å) protruding from loop β3-α3. In the obtained crystal form, His35 from another MtrA molecule of the asymmetric unit occupied the sixth ligation site of cobalt, which resulted in a hexacoordinated Co(III) oxidation state (see Fig. 2a). In addition, two propionamide substituents of the corrinoid ring were hydrogen bonded with Asn82-NH or bidentately bonded with the Asn55 amide (the latter was observed only in two molecules of the asymmetric unit) (Supplementary Fig. S8).

The nucleotide tail was accurately embedded into a dominantly hydrophobic pocket lined with β0, β1, β2, helix α2, loop β2-α2 and the N-terminal segment (Fig. 4a) and firmly fixed by multiple hydrophobic interactions and specific hydrogen bonds to Asp17-N, Lys52-N, Thr53-OG1 and -N, Gly57-O, and Asn64-ND2. The dimethylbenzimidazole group was sandwiched between the hydrophobic side chains of Tyr18 and Val61. Most residues involved in cobalamin binding were fully conserved in the two MtrA variants (Supplementary Figs S9 and S10).

Superposition studies surprisingly revealed a new binding site for cobalamin in MtrA compared with all other Rossmann-type B_12_ enzymes whose corrinoid rings cluster around the same position (Supplementary Fig. S11). The corrinoid rings are surrounded by different loops and the nucleotide tails bind at different sides of the central β-sheet. Consequently, none of the cobalamin–polypeptide interactions is conserved between MtrA and all other Rossmann-type B_12_ enzymes. Methionine synthase (MetH) is a typical representative of the already established cobalamin binding mode (Figs 4b and 5).

Despite their different environments, the elongated cobalamin conformation is similar in MtrA and the other Rossmann-type B_12_ enzymes and is clearly distinguished from the conformation of base-on/His-off cobalamins i.e., dioldehydratase^23^,^24^ and ribonucleotide reductase^27^ and those that are more compact and curled, i.e., reductive dehalogenase^22^. Apparently, B_12_ enzymes (but not B_12_-trafficking and membrane transport proteins) with an elongated cobalamin contain a Rossmann-type domain^15^, which suggests that functional constraints favour a specific fold to a certain extent. Despite their shared overall conformation, the cobalamins of the Rossmann-type B_12_ enzymes vary in the position or orientation of the ring propionamide and acetamide substituents and of the dimethylbenzimidazole base (Supplementary Figs S11 and S12).

An analysis of the alternative binding modes of cobalamin indicated that each of the two binding sites was installed into a Rossmann-type scaffold by a different construction of the loops at the C-terminal end of the parallel β-sheet. MtrA has no overall sequence similarity to other B_12_-binding proteins, which indicated that the ancestral gene of MtrA appears to be different from those of other B_12_-binding proteins. Evolution, starting from two different ancestral genes, independently came up with highly equivalent solutions with respect to a cobalamin in an elongated, mostly His-on, conformation with the nucleotide tail embedded between the central β-sheet and an α-helix and with respect to an exposed corrinoid ring with loose contacts to the polypeptide.

### Cobalt-ligation and energy propagation

Despite the newly constructed cobalamin-binding site found in MtrA, the basic interaction network of the lower axial ligand of cobalt, in principle, corresponds to that found for other Rossmann-type B_12_ enzymes, with the exception of the corrinoid iron-sulphur domains. In MtrA, His84 pointing from a protrusion of loop β3-α3 towards the corrinoid ring, was coordinated to Co(III) via NE2 and was hydrogen bonded via ND1 to Glu54-OE1. Glu54 projecting from loop β2-α2 further interacted via OE1 and OE2 to Glu54-N and Ile111-N, respectively, which mutually fixes the position of the carboxylate and the interacting loops (Fig. 5). Ile111 is a residue of the pronounced meander-like segment following helix α3 (Supplementary Fig. S13). In methionine synthase, methanol cobalamin methyltransferase, and methylmalonyl-CoA mutase, a functionally analogous glutamate or aspartate was identified that was hydrogen bonded with a proton-donating serine, threonine and peptide amide, respectively^16^,^20^, albeit in completely different polypeptide environments (see Fig. 4b for methionine synthase). As determined for methionine synthase, the catalytic His-Asp-hydrogen-bonding donor linkage stabilizes the Co(III) state via histidine protonation and controls conformational signalling^16^.

**Figure 1:**
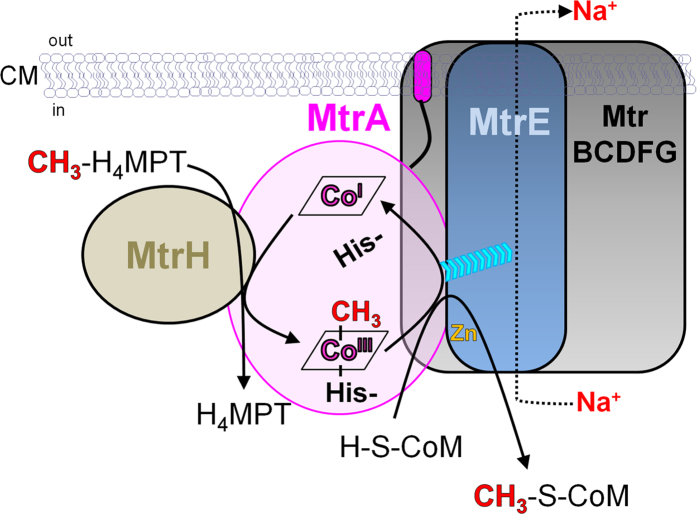
Organization and catalytic cycle of the MtrA–H complex. In a two-step reaction, the methyl group of methyl-tetrahydromethanopterin (CH_3_-H_4_MPT) bound to MtrH is first transferred to cobalamin bound to MtrA and second from there to coenzyme M (CoM-SH). It is postulated that the latter methyl-transfer reaction provokes sodium-ion translocation localized on MtrE^1^. The cyan arrowheads indicate energy transfer from the displaced histidine to MtrE upon its dissociation from Co(I). The sketched size of the subunits does not correspond to their molecular masses. CM: cytoplasmic membrane.

**Figure 2:**
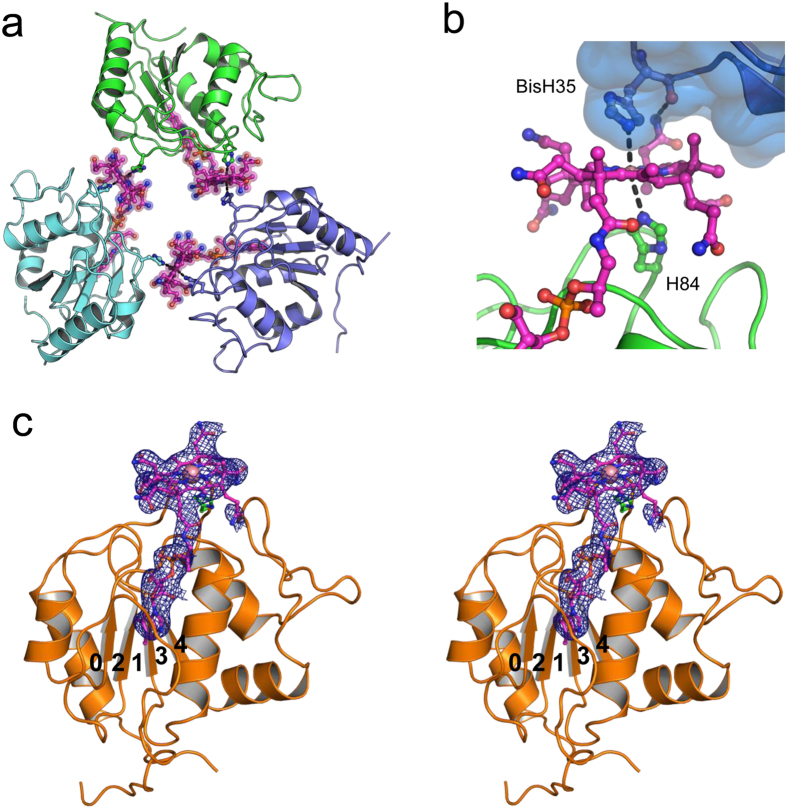
Structure of the cytoplasmic MtrA homologue from *M. fervidus*. (**a**) Triangular arrangement of the three MtrA homologues (green, blue, cyan) in the asymmetric unit. Cobalamin, the lower axial ligand (His84) and the artificial sixth axial ligand (His35 from the neighbouring MtrA) are shown as ball and stick models (carbons for B_12_ in pink). (**b**) The bisHis ligation structure of the cobalt is shown in detail. A significant contact area between the subunits is absent. (**c**) Rossmann-type fold of the MtrA homologue. The 2*F*_o_-*F*_c_ map at the cobalamin molecule is contoured at 1.0 σ (blue mesh).

**Figure 3:**
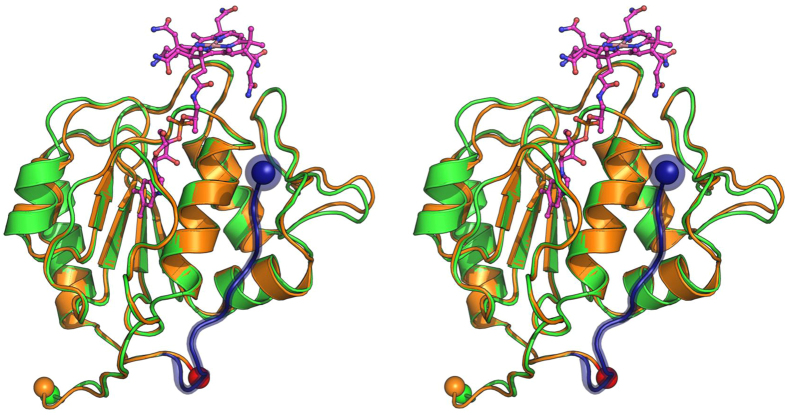
Structure of the soluble domain of the membrane-associated MtrA from *M. jannaschii*. The soluble domain of membrane-associated MtrA from *M. jannaschii* (green) and the cytoplasmic MtrA homologue from *M. fervidus* (orange) were superimposed. Their N-termini are shown as orange and green spheres, and their C-termini as dark-blue and red spheres, respectively. The juxtamembrane segment of MtrA from *M. jannaschii* is drawn in dark-blue, the cobalamin of the MtrA homologue is drawn as a pink stick model.

**Figure 4:**
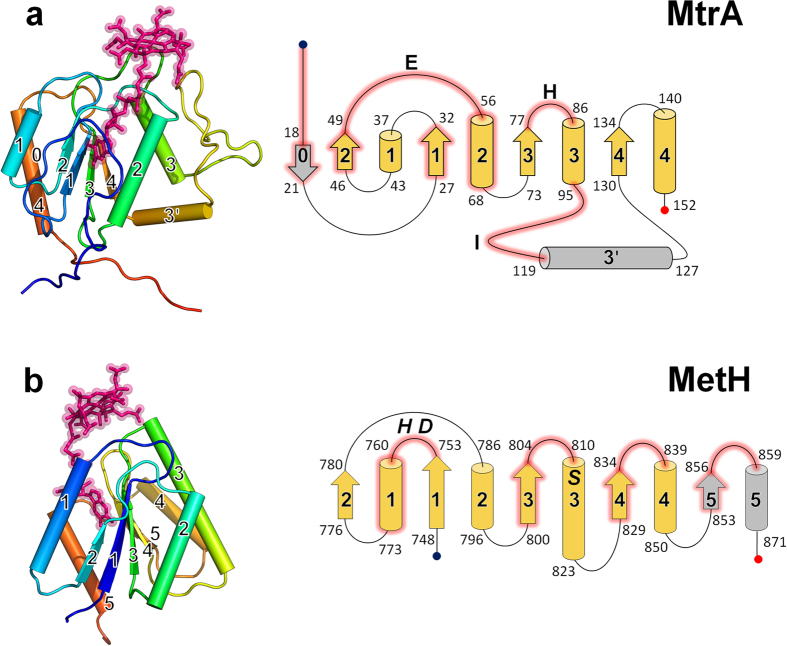
Folds and cobalamin binding of (**a**) the cytoplasmic MtrA homologue of *M. fervidus* and (**b**) MetH of *E. coli*. (Left) Three-dimensional secondary structures represented with α-helices as solid tubes, β-sheets as arrows, and loops as strings. The cobalamin-binding pockets in MtrA and MetH are located at opposite side of the central β-sheet. Cobalamin is shown in pink. (Right) Topology diagram with the regions forming the cobalamin binding pocket in pink and the residues of the hydrogen-bonding linkage as single-letter code. Rossmann-type B_12_ domains including MtrA and MetH share the core topology motif β2α1β1α2β3α3β4α4 highlighted in yellow. The secondary structures are named by the numbers of their first and last amino acids.

**Figure 5:**
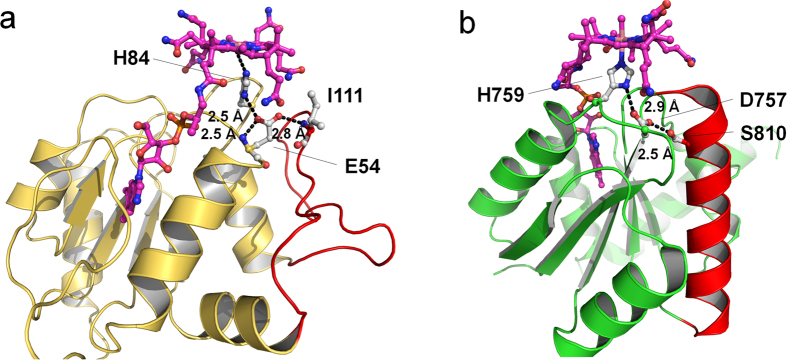
The hydrogen-bond linkage and its interactions to the polypeptide surrounding. (**a**) The cytoplasmic MtrA homologue-cobalamin complex from *M. fervidus* is shown. The hydrogen-bond linkage composed of His84, Glu54 and Ile111 links the cobalt of cobalamin and the meander-like structure (red). (**b**) The methionine synthase MetH-domain-cobalamin complex from *E. coli* also contains a cobalamin and the hydrogen-bond linkage consisting of His759, Asp757 and Ser810. Ser810 is located at near the N-terminal end of helix α3^16^ (red).

According to current opinion, the sodium ion translocation process is induced by the demethylation of methyl-cob(III)alamin to cob(I)alamin^1^. This reaction results in a repulsion between the electron-rich species Co(I) and His-imidazolium^1^,^13^,^29^. The accompanied displacement of the His-imidazolium is specifically propagated to adjacent polypeptide segments and from there to MtrE (see Fig. 1 and Supplementary Fig. S2). We have determined the structure of MtrA in a cobalamin-free and cob(III)alamin-bound form, both representing relaxed conformations, but not in a strained cob(I)alamin form. Although our experimental data do not allow for deeper mechanistic insights, the protrusion after helix α3 and the adjacent insertion region (Figs 4a and 5) are structural elements susceptible to conformational changes. In particular, the unique meander-like segment of the insertion region has only a few contacts to the protein core but is strongly connected to His84 (via Glu54) (Supplementary Figure S13), which might oscillate between two positions depending on the oxidation state of cobalt^1^. This surface region is relatively mobile and deviates between the two MtrA variants more than most other regions. Together with the loops β2-α2 and β3-α3, which contain Glu54 and His84, respectively, the meander-like segment is the most conserved region in MtrA and even allows for the identification of the consensus sequence ^109^GAIPF-Y/F-XEN (X: hydrophobic amino acids), which emphasizes its importance. The structural rearrangement could possibly be detected in the structure of a His-unligated Co(I) state, but this structure is not easily obtainable.

## Conclusion

Crystal structures of two MtrA variants revealed a detailed view of a novel binding site for cobalamin inside a Rossmann-type domain compared with all other Rossmann-type B_12_ enzymes studied so far. Nevertheless, several functionally characteristic features were designed in an equivalent manner. They include an exposed corrinoid ring at the top of loops at the C-terminal side of the central β-sheet, an elongated conformation of cobalamin with the nucleotide tail between the β-sheet–α-helix layer, albeit on opposite sides, and a His-Glu-proton donor linkage on the lower axial side of the corrinoid ring.

The structure of cytoplasmic MtrA complexed with cobalamin and the structure of the soluble domain of MtrA without cobalamin were essentially identical. This finding suggested that the MtrA structures observed in this work were relaxed forms. The possible repellent effect of cob(I)alamin towards the histidine residue probably causes a conformational change in the MtrA protein structure (Fig. 1 and Supplementary Fig. S2)^1^,^13^,^29^. From there, the conformational changes could be transmitted further for sodium-ion transport across the cytoplasmic membrane.

## Materials and Methods

### Bioinformatics searches for the cytoplasmic MtrA homologues

Membrane-associated MtrA from *M. marburgensis* was used as a search query for a general basic local alignment search tool (BLAST) of the NCBI protein database. The discrimination criteria between membrane-associated MtrA and cytoplasmic MtrA homologue were mainly based on the sequence length and the prediction of the membrane segment using the Octopus server^30^; sequences less than 215 amino acids correspond to cytoplasmic MtrA homologue without the juxtamembrane and transmembrane segments (Supplementary Figs S1 and S5).

### Preparation of MtrA proteins

The pET28a expression vector, containing DNA fragments for heterologous expression, was purchased from GenScript (Piscataway, New Jersey, United States); the codon usage of constructs was optimized for expression in *E. coli*. Sequences encoding MtrA were inserted into the expression vector (those of the soluble domain of membrane-associated MtrA from *M. jannaschii* and cytoplasmic MtrA homologue from *M. fervidus* are shown in Supplementary Fig. S5). *E. coli* BL21(DE3) was transformed with the recombinant plasmids. Cells were grown at 37 °C in Luria Bertani (LB) medium supplemented with 50 μg/ml of kanamycin. When the OD_600_ reached 0.6, 1 mM of isopropyl β-d-thiogalactopyranoside (Roth, Karlsruhe, Germany) was added to induce gene expression. After a 3 h incubation, cells were harvested, washed with 8 mM of phosphate buffer, pH 7.4 supplemented with 150 mM NaCl and 3 mM KCl, and stored at −80 °C for further use.

For expression, we tested gene constructs of the soluble domain of membrane-associated MtrA from the methanogens *M. marburgensis*, *M. evestigatum*, *M. kandleri* and *M. jannaschii*. All genes were highly expressed in *E. coli* BL21(DE3), and a soluble protein was synthesized. The cytoplasmic MtrA homologues from *M. fervidus* and *M. evestigatum* were expressed, but only the protein from *M. fervidus* was obtained in a soluble form in *E. coli* BL21(DE3).

All procedures for purification of MtrA constructs were performed on ice or at 4 °C. Frozen cells containing MtrA were re-suspended in 50 mM of sodium phosphate buffer pH 8.0 containing 0.5 M NaCl, 25 mM imidazole and 5% glycerol (buffer A) and disrupted by sonication (Bandelin Electronic, Berlin, Germany) using a VS70 tip with 50% power, seven times for 2 min with 5 min breaks. The lysate was centrifuged at 30,000 × *g* for 45 min at 4 °C. The supernatant was filtered through a 0.45 μm filter and loaded onto a Ni^2+^ -charged HiTrap chelating column (GE Healthcare, Uppsala, Sweden) equilibrated with buffer A. The column was washed extensively with buffer A, and protein was eluted with a linear gradient of imidazole from 25 to 1,000 mM. Fractions were analysed by SDS-PAGE; fractions containing purified MtrA were pooled and concentrated to 1 ml with a 3 kDa cut-off centrifugal filter unit (Millipore).

### Cobalamin incorporation

Although methanogenic archaea without cytochromes, including *M. jannaschii* and *M. fervidus*, contain 5-hydroxybenzimidazolyl cobamide (factor III) rather than 5-dimethylbenzimidazolyl cobamide (cobalamin)^31^, cobalamin was used for the reconstitution experiments^11^. The use of cobalamin instead of factor III, which is commercially unavailable, was possible because the Mtr complex of the cytochrome-free *M. marburgensis*, grown in the presence of dimethylbenzimidazole, was active^11^,^31^. All studied MtrA variants were unfolded as follows: 544 μl of 8.0 M guanidinium hydrochloride pH 7.0, 175 μl of 0.5 M 3-(*N*-morpholino)propanesulphonic acid (MOPS) pH 7.0 and 17.5 μl of 1 M dithiothreitol (DTT) were added to 1 ml of a concentrated protein solution. The proteins unfolded at room temperature within at least 2 h. Refolding proceeded in an anoxic tent at room temperature by adding a solution of unfolded protein dropwise into a 25-fold volume of 25 mM Tris-HCl pH 7.6 (44 ml), 150 mM NaCl, 2 mM DTT, 5% glycerol and 0.5 mM methylcobalamin or hydroxocobalamin with gentle stirring to avoid local unspecific aggregation^11^. In addition, to cleave the His-tag of the cytoplasmic MtrA homologue from *M. fervidus*, thrombin (Sigma-Aldrich, Taufkirchen, Germany) was supplemented to the refolding solution (1.4 mg/ml final concentration). Refolding occurred overnight in an amber glass bottle as cobalamin is light sensitive. All subsequent steps were performed under red light to protect the cobalamin.

We tested the stability of the protein−cobalamin complex by gel filtration. First, the protein refolded with cobalamin was centrifuged under air at 30,000 × *g* for 30 min at 4 °C to remove unfolded protein aggregates. The supernatant was filtered through a 0.45 μm filter and loaded onto a Ni^2+^ -charged HiTrap chelating column. The column was extensively washed with 25 mM Tris-HCl pH 7.6 containing 150 mM NaCl, 2 mM DTT and 5% glycerol (GF buffer) to remove unbound cobalamin. Protein was eluted with GF buffer containing 500 mM imidazole and subsequently concentrated in a 3 kDa cut-off centrifugal filter unit. In the case of the cytoplasmic MtrA homologue from *M. fervidus*, cobalamin and the cleaved His-tag segment were removed by concentration and dilution via ultrafiltration (3-kDa cut-off). The MtrA proteins were then injected onto a HiPrep Sephacryl S100 16/60 (GE Healthcare, Freiburg, Germany) column equilibrated with GF buffer. The amount of cobalamin loaded on the protein sample was estimated from the UV/Vis spectrum (SPECORD S600 Analytik Jena, Jena, Germany). The soluble domains of the membrane-associated MtrA homologues from *M. marburgensis* and *M. kandleri* have less than 40% cobalamin. The soluble domain of membrane-associated MtrA from *M. evestigatum* appeared to contain unspecifically bound cobalamin, as the visible region corresponding to cobalamin had an extremely high absorbance. The only MtrA protein with an approximately 1:1 cobalamin:protein ratio was the soluble domain of the membrane-associated MtrA from *M. jannaschii* and the cytoplasmic MtrA homologue from *M. fervidus.* Therefore, we used these MtrA proteins for crystallization.

Purity of the proteins was controlled by SDS-PAGE. The protein concentration was determined using the Bradford method with the dye reagent and bovine serum albumin from Bio-Rad.

### Limited proteolysis by trypsin

Trypsin (Sigma-Aldrich) was added at a trypsin:MtrA ratio of 1:2,000 (w/w) to a solution of refolded cobalamin-bound soluble domain of membrane-associated MtrA from *M. jannaschii* in the presence of 10 mM CaCl_2_ (final concentration) and incubated for 6 min at 30 °C. Proteolysis was stopped by adding the irreversible trypsin inhibitor, *N*_α_-tosyl-l-lysine chloromethylketone (0.2 mM; Sigma-Aldrich). The cleaved protein was injected onto a HiPrep Sephacryl S100 16/60 column equilibrated with GF buffer. The eluted protein was analysed by SDS-PAGE.

### Protein crystallization

The cytoplasmic MtrA homologue from *M. fervidus*, in complex with either hydroxocobalamin or methylcobalamin, was crystallized using 20 mg/ml protein solution (in GF buffer) under oxic conditions and red light at room temperature. After several months, the hydroxocobalamin complex developed two different crystalline forms, one pink and one orange. Both forms diffracted to a maximum of ca. 6 Å resolution and were therefore not suitable for structural analysis. After approximately one year, only one pink crystal in a new crystalline form appeared in 26% PEG 3000, 100 mM Na citrate pH 5.5. The crystallization drop was prepared by mixing protein and precipitant at a 1:1 ratio using the vapour diffusion method with 1 μl of protein solution (CombiClover Junior Plate, Jena Bioscience, Jena, Germany) and 100 μl of reservoir solution. This crystal diffracted to 3.0 Å. The soluble domain of the membrane-associated MtrA from *M. jannaschii* in the presence of either methylcobalamin or hydroxocobalamin (cleaved by limited proteolysis) was crystallized at 50 mg/ml at 8 °C under oxic conditions and red light. After 2 months of incubation, transparent needle-type crystals were reproducibly obtained in 2.2 M d/l-malate pH 7.6, 100 mM Tris-HCl pH 8.0 under the conditions described above.

### Data collection and structural analysis

Crystals of the cytoplasmic MtrA homologue from *M. fervidus* and crystals of the soluble domain of membrane-associated MtrA from *M. jannaschii* were cryoprotected in 25% glycerol and flash-frozen in liquid nitrogen. Diffraction experiments were conducted at 100 K on the beamline X10SA equipped with a PILATUS 6 M detector at the Swiss Light Source synchrotron (Villigen). Data were processed with iMOSFLM^32^. Data of the cleaved soluble domain of the membrane-associated MtrA of *M. jannaschii* were scaled with SCALA, and those of the cytoplasmic MtrA homologue from *M. fervidus* were scaled with AIMLESS in the ccp4 suite^33^. The high symmetry space group of the crystal of the cytoplasmic MtrA homologue from *M. fervidus* helped to solve the structure (Supplementary Table 1, I422 space group) using the anomalous signal of cobalt from cobalamin. Single anomalous dispersion (SAD) data were measured at the cobalt K edge. Cobalt atom sites were localized with SHELXC/D^34^. Phase determination and electron density modification were conducted with the program AutoSol from the PHENIX package^35^. The model was built using automated AutoBuild from the PHENIX package^35^. The crystal structure of the soluble domain of the membrane-associated MtrA from *M. jannaschii* was solved by molecular replacement using Molrep from the ccp4 suite^33^. The structure of the cytoplasmic MtrA homologue from *M. fervidus* was used as a search model. Both models were then manually improved with COOT^36^. The model of cytoplasmic MtrA from *M. fervidus* was further refined with REFMAC5^37^ and that of the soluble domain of membrane-associated MtrA from *M. jannaschii* was refined with PHENIX^35^. The structure of MtrA homolog from *M. fervidus* contains the cobalamin. Since the crystallographic data for this structure is about 3.0 Å, we impose high geometry restraints to maintain the corrinoid planarity. In a first step, the B_12_ library was uploaded in Jligand from the ccp4 package and save as a “.cif” file. This file was manually edited to increase the planarity restraint. We use this new library to keep going the refinement using REFMAC5. Non-crystallographic symmetry (NCS) restraints and translation-libration-screw-rotation (TLS)^37^ were applied to both models. Final models were validated using the MolProbity server (http://molprobity.biochem.duke.edu)^38^. Data quality and refinement statistics are listed in Supplementary Table 1. Figures were generated with PyMOL (Version 1.5, Schrödinger, LLC). The MtrA sequence was aligned with sequences of related proteins from other methanogens using the ClustalW2 server (http://www.ebi.ac.uk/Tools/msa/clustalw2/), secondary structures were aligned using ESPript v 3.0 (http://espript.ibcp.fr/ESPript/ESPript/)^39^ and tertiary structures were superimposed using the CONSURF server (http://consurf.tau.ac.il/)^40^ or the Superpose program of the ccp4 suite^33^. The folds of MtrA and MetH family proteins (Fig. 4) were compared using the Pro-Origami server (http://munk.csse.unimelb.edu.au/pro-origami/)^41^.

## Additional Information

**How to cite this article**: Wagner, T. *et al*. MtrA of the sodium ion pumping methyltransferase binds cobalamin in a unique mode. *Sci. Rep.* **6**, 28226; doi: 10.1038/srep28226 (2016).

## Supplementary Material

Supplementary Information
