# Supplementary material for: MtrA of the sodium ion pumping methyltransferase binds cobalamin in a unique mode
Source: Sci Rep. 2016 Jun 21;6:28226. doi: 10.1038/srep28226 (PMC4915002; doi:10.1038/srep28226)
Supplement: Supplementary Information [file srep28226-s1.pdf]

Supplementary Information

MtrA of the sodium ion pumping methyltransferase binds cobalamin in a unique mode

Tristan Wagner[1], Ulrich Ermler[2] and Seigo Shima[1,3*]

[1]Max Planck Institute for Terrestrial Microbiology, Karl-von-Frisch-Straße 10, 35043 Marburg, Germany. [2]Max Planck Institute for Biophysics, Max-von-Laue-Straße 3, 60438 Frankfurt am Main, Germany. [3]PRESTO, Japan Science and Technology Agency (JST), 4-1-8 Honcho Kawaguchi, 332-0012 Saitama, Japan.

*Correspondence and requests for materials should be addressed to S.S. (e-mail: shima@mpi-marburg.mpg.de).

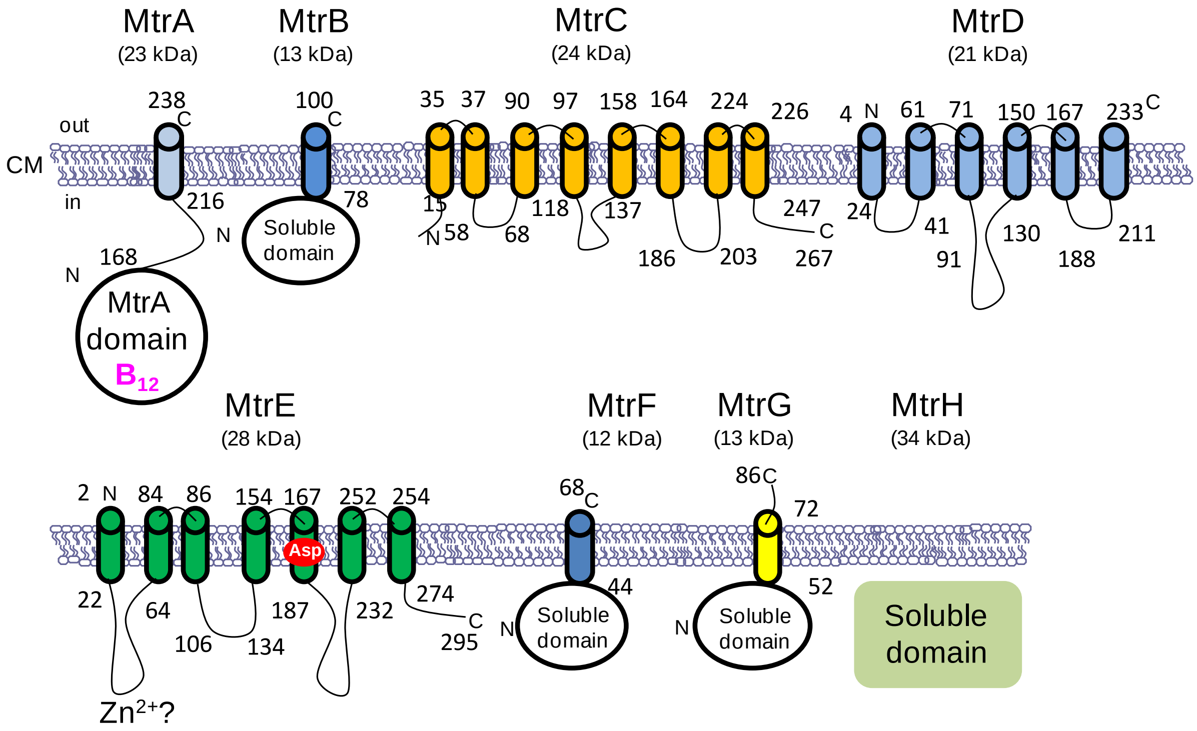

Supplementary Figure S1. Topology of the eight subunits of MtrA–H. The amino acid numbers indicated are taken from the membrane-associated sodium-ion-translocating methyltransferase complex from *M. marburgensis*. The cytoplasmic domains and the loops are not drawn to scale relative to the transmembrane helices. CM, cytoplasmic membrane; C, C-terminal end; N, N-terminal end (Figure modified from Ref. 1).

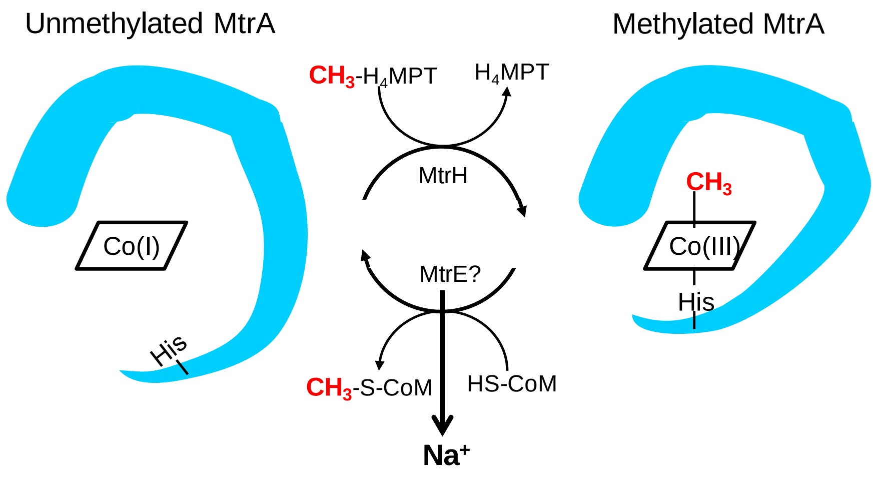

Supplementary Figure S2. Proposed conformational change of MtrA associated with methylation and demethylation of its corrinoid prosthetic group. The coordination chemistry of cobalamin predicts that in the reduced non-methylated Co(I) form, the histidine ligand will be base-off, and in the methylated Co(III) form it will be base-on (Figure modified from Ref. 1). The cyan regions indicate the part of the MtrA protein involved in coordination of cobalamin.

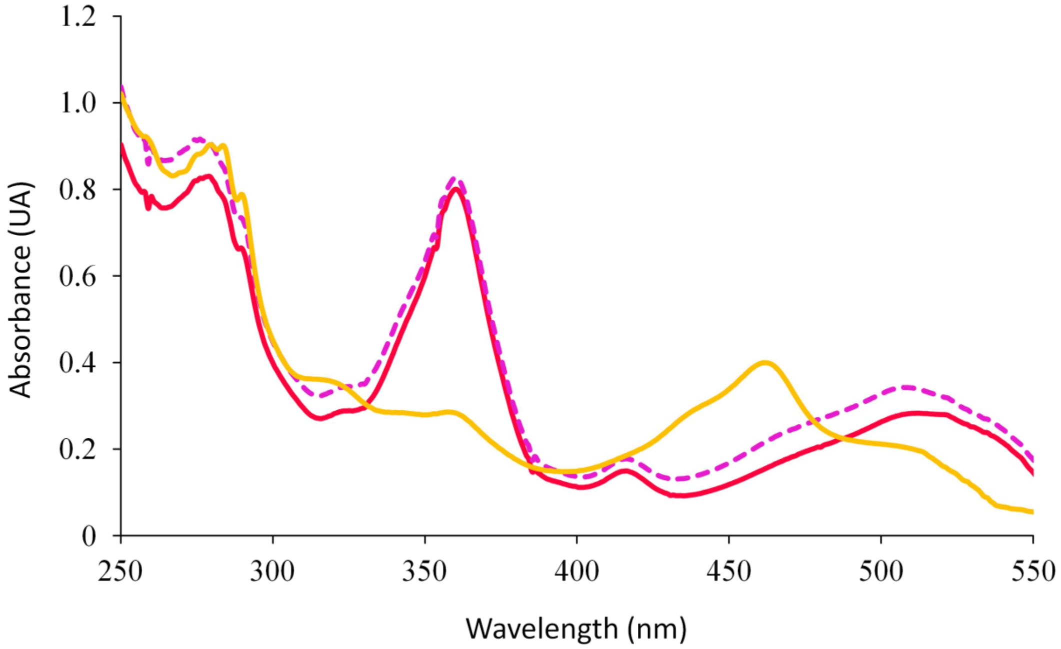

Supplementary Figure S3. UV-Vis spectra of the cytoplasmic MtrA homolog from *M. fervidus* after reconstitution. Red line: protein just after reconstitution with methyl-cobalamin (CH_3-B_{12}) which indicated the Co(III) state; this spectrum did not change after 1 day of incubation. Pink dashed line: protein after reconstitution with hydroxocobalamin (OH-B_{12}), which indicated the Co(III) state. Orange line: protein reconstituted with hydroxocobalamin (OH-B_{12}) after 1 day of incubation at 4 °C, which indicated the Co(II) state.

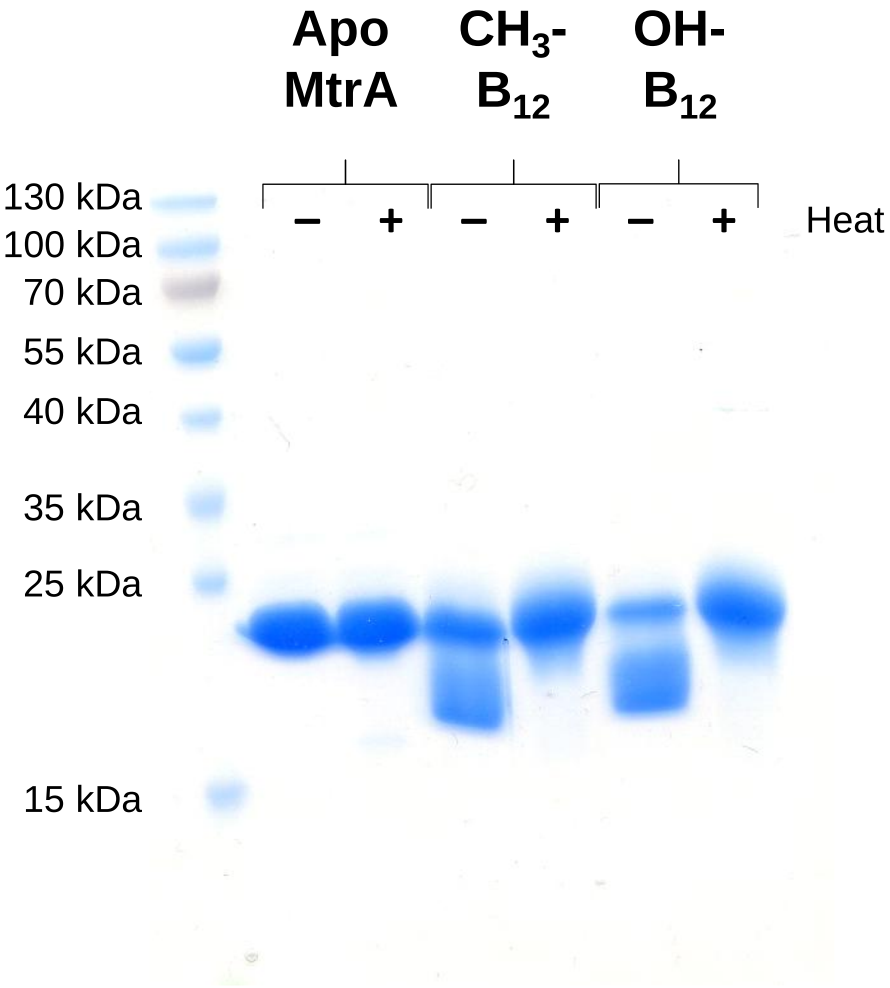

Supplementary Figure S4. SDS-PAGE of the cytoplasmic MtrA homolog from *M. fervidus* reconstituted with cobalamin. ApoMtrA, MtrA without cobalamin; CH_3-B_{12}, the cytoplasmic MtrA homolog reconstituted with methyl-cobalamin; OH-B_{12}, the cytoplasmic MtrA homolog reconstituted with hydroxo-cobalamin. The samples were prepared in standard buffer containing 1% SDS as described by Laemmli[41] with (+) or without (–) heating at 95 °C for 5 min. Protein was stained with Coomassie dye solution (InstantBlue, Expedion).

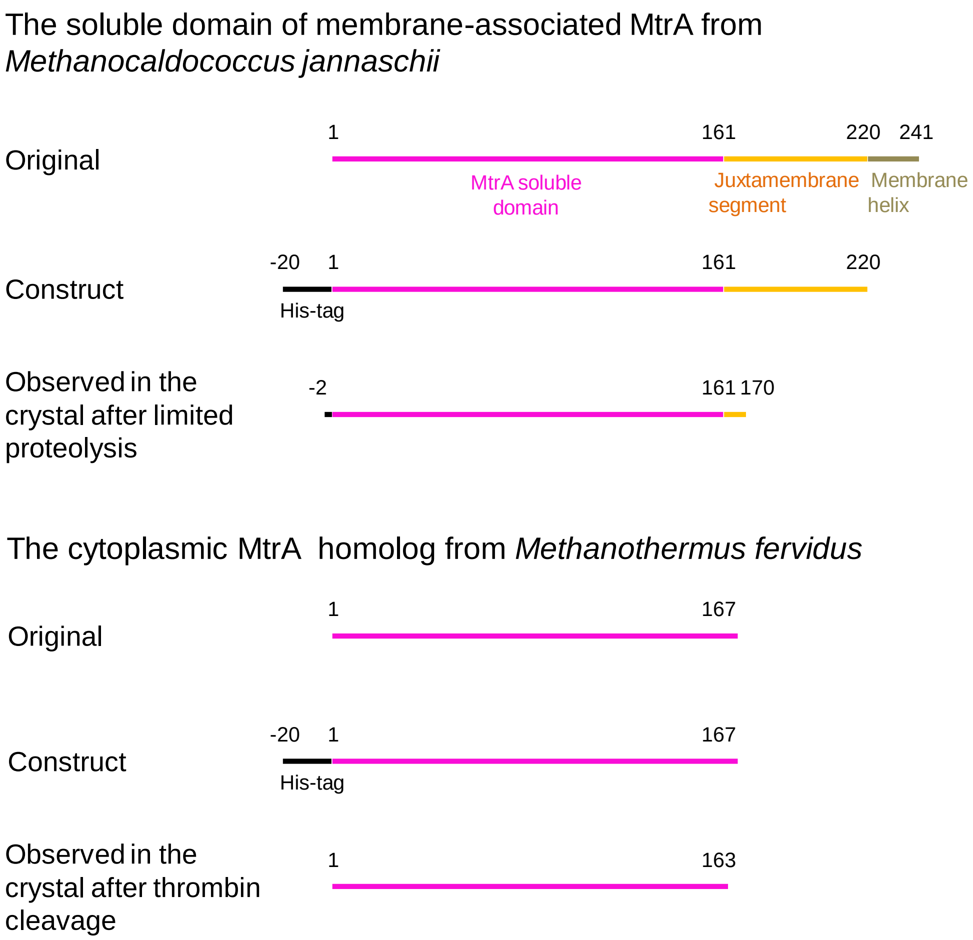

Supplementary Figure S5. Gene constructs used in this study. The gene of the soluble part of membrane-bound MtrA from *M. jannaschii* was obtained from the original amino-acid sequence encoded in the genome. The gene was chemically synthesized in a manner, that the codons are optimized for expression in *E. coli* and cloned into pET28a vector. The protein produced was partially proteolyzed and crystallized as described in the Method section. The amino-acid sequence (1-167) of the cytoplasmic MtrA homolog from *M. fervidus* corresponds to that encoded in the genome. The DNA fragments were inserted into vector pET28a at the *Nde*I and *Eco*RI cleaving sites such that an N-terminal His-tag was fused. The synthesized genes contain *Nde*I and *Eco*RI sites at the 5′ and 3′ ends, respectively. The primary structure of the original gene sequences, the construct in the expression vectors and the amino acid chain visible in the electron density after limited proteolysis prior to crystallization are indicated.

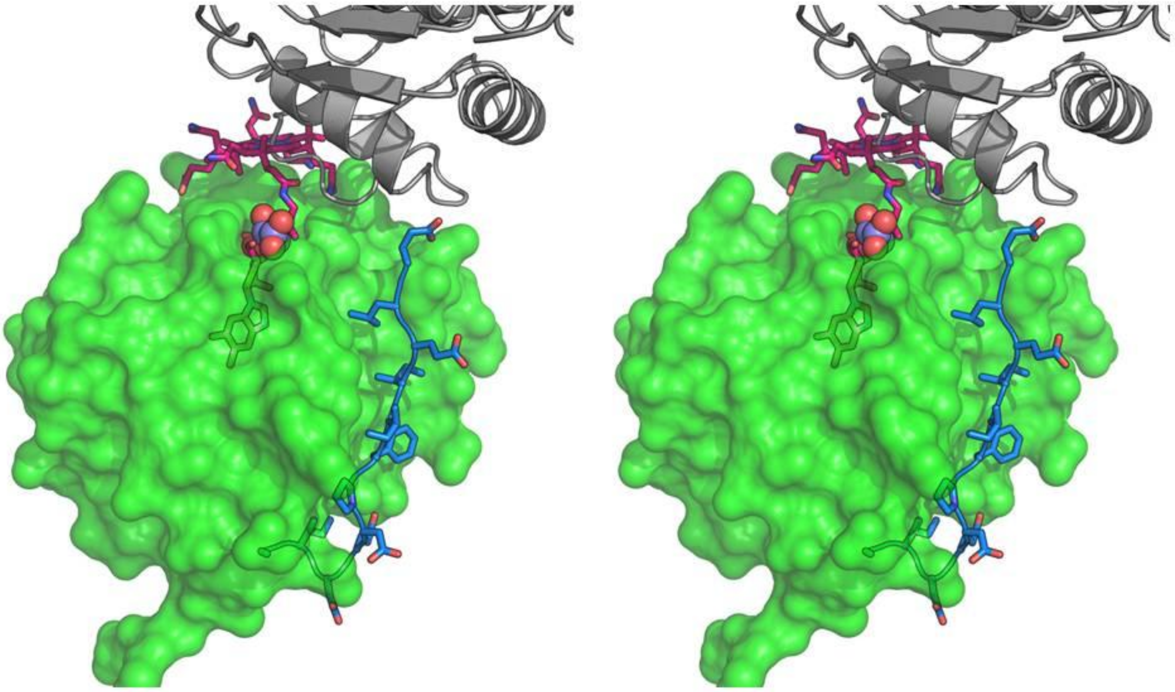

Supplementary Figure S6. Structure of the soluble domain of membrane-associated MtrA from *M. jannaschii*. a) One monomer in the asymmetric unit is shown as a green surface model; a part of the second monomer is shown in grey. The juxta-membrane segment is shown as a blue stick model. Cobalamin (pink stick model) was modelled by superimposing the cytoplasmic MtrA homolog onto the soluble domain of membrane-associated MtrA. In the crystal form of the latter cobalamin would clash with the other molecule of the crystal lattice (grey). A malate molecule bound to the protein is represented by spheres.

a

Pyruvate dehydrogenase

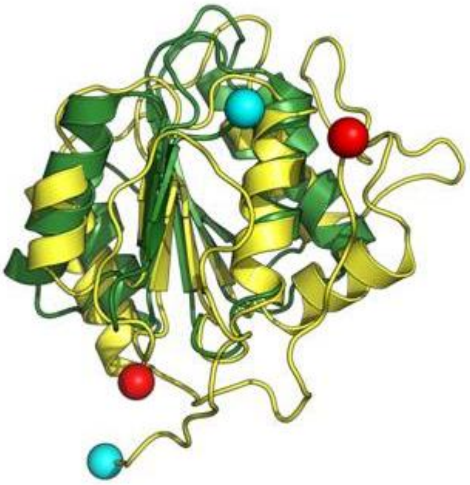

Succinyl-CoA synthase

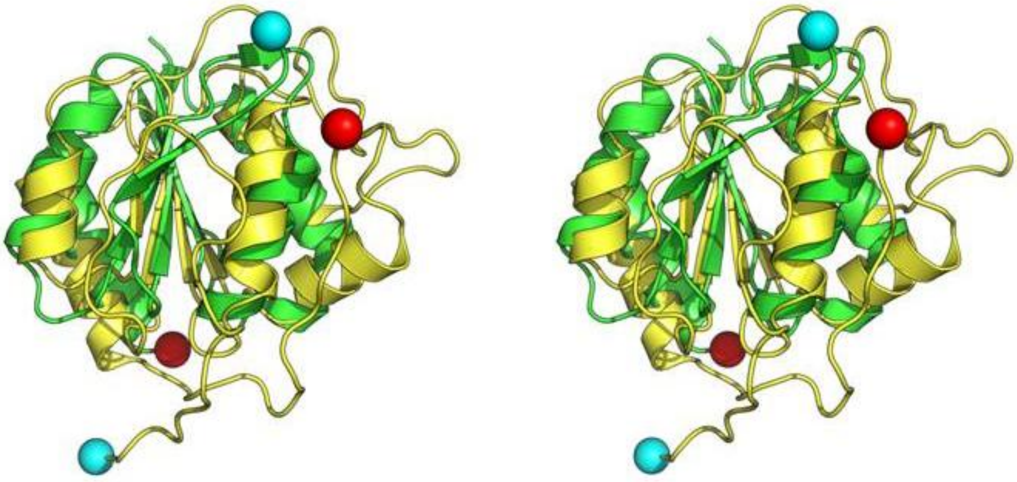

b

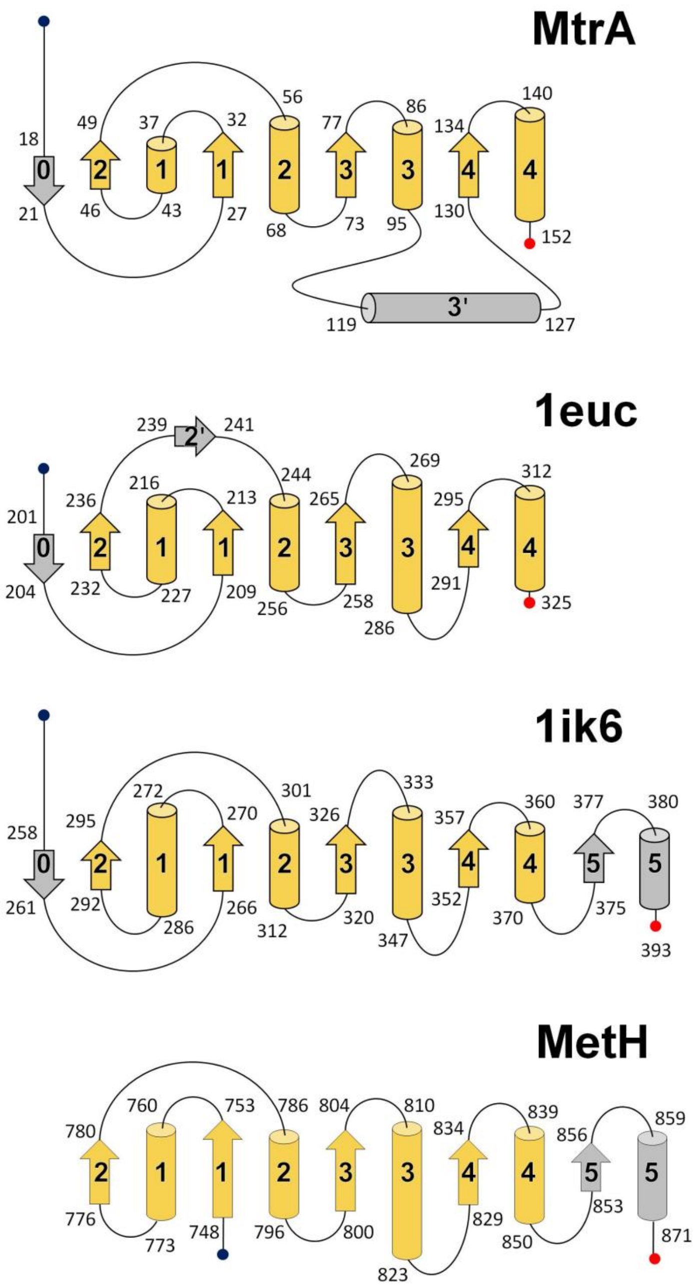

Supplementary Figure S7. Superposition of MtrA with its structural homologues and the topology diagrams. a) Superposition of the cytoplasmic MtrA homolog from *M. fervidus* with pyruvate dehydrogenase from *Pyrobaculum aerophilum* (PDB code: 1ik6) or succinyl-CoA synthetase from *Sus scrofa* (wild boar) (PDB code: 1euc). The N- and C-termini of the proteins are shown as red and cyan spheres. **b**) Topology diagram of the protein structures.

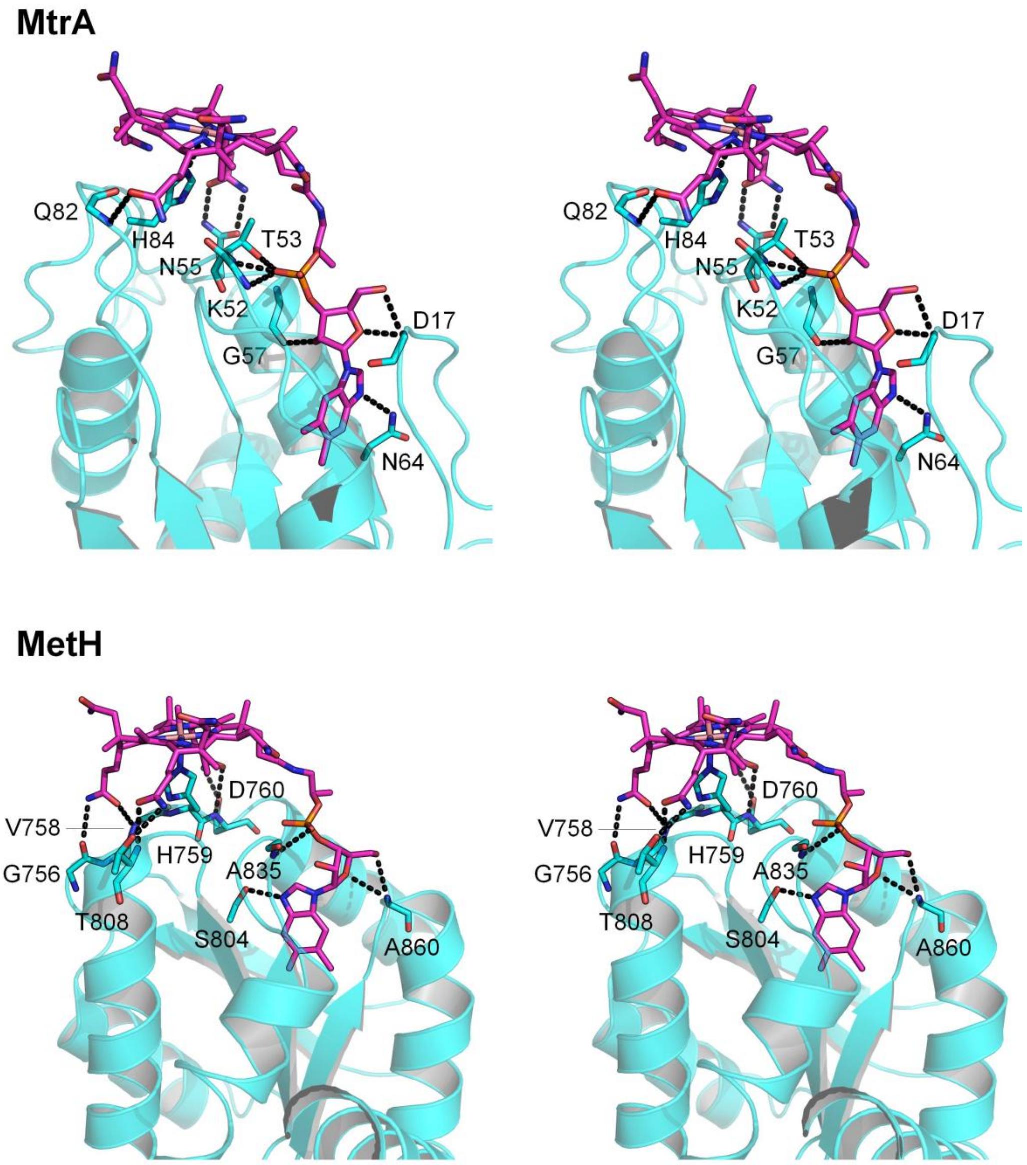

Supplementary Figure S8. Comparison of the cobalamin binding interface of the cytoplasmic MtrA homolog from *M. fervidus* and MetH from *E. coli*. Cobalamin (pink) and the amino acid residues involved in hydrogen bonding (orange) are depicted as stick models. MetH contains the following consensus sequence for binding of cobalamin: ^{757}DX^{759}HXX^{762}G–^{804}SXL–^{833}G^{834}G. His759 is the lower axial ligand, and Asp757 hydrogen bonds to His759. In MtrA His84 and Glu54 are found at the equivalent positions of the three-dimensional structures. Gly762 of MetH locates near the phosphate-O; at the same position of both MtrA structures, Gly57 is found. The position of Ser804, Leu806, Gly833 and Gly834 was occupied with other amino acid residues in MtrA. The place of Gly833 and Gly834 was occupied with Val61 and Lys60 in MtrA, respectively. However, the space between dimethylbenzimidazole and the main chain part was almost the same in MtrA and MetH.

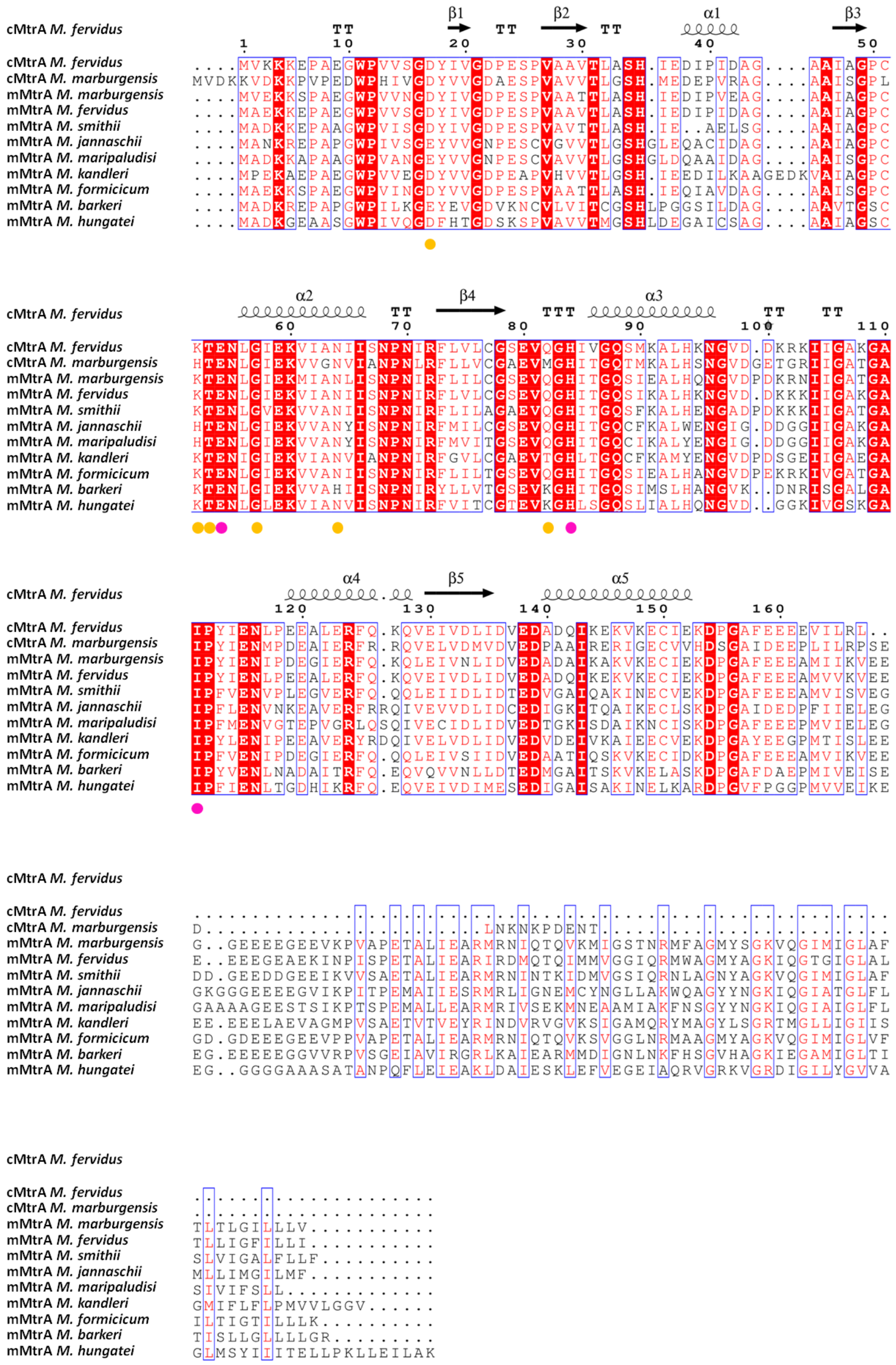

Supplementary Figure S9. Sequence alignment of MtrA proteins from different methanogens. cMtrA, the cytoplasmic MtrA homologs; mMtrA, membrane-associated MtrA. Full species names: *M. fervidus*, *Methanothermobacter marburgensis*, *Methanobrevibacter smithii*, *M. jannaschii*, *Methanococcus maripaludis*, *Methanopyrus kandleri*, *Methanobacterium formicicum*, *Methanosarcina barkeri*, *Methanospirillum hungatei*. Orange dots, residues involved in polar contacts with cobalamin; pink dots, residues involved in the hydrogen-bonding network of the lower axial ligand of cobalamin. The secondary structures of the cytoplasmic MtrA homolog are sketched in. TT indicates turn structure.

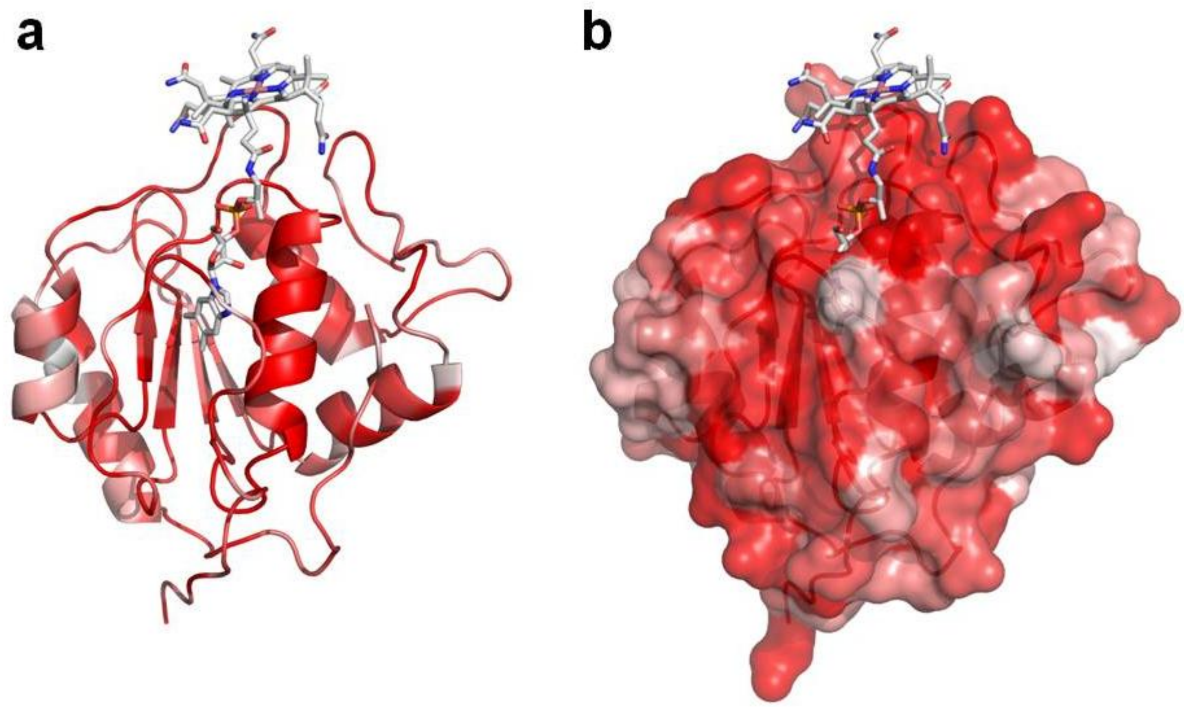

Supplementary Figure S10. 3D representation of the conserved region of the cytoplasmic MtrA homolog from *Methanothermus fervidus*. a) Cartoon and **b**) surface models are coloured according to the conservation score taking into account the sequence alignment of the chosen MtrA variants (Supplementary Fig. S9): white, no conservation; light red, partial conservation; and red, strict conservation.

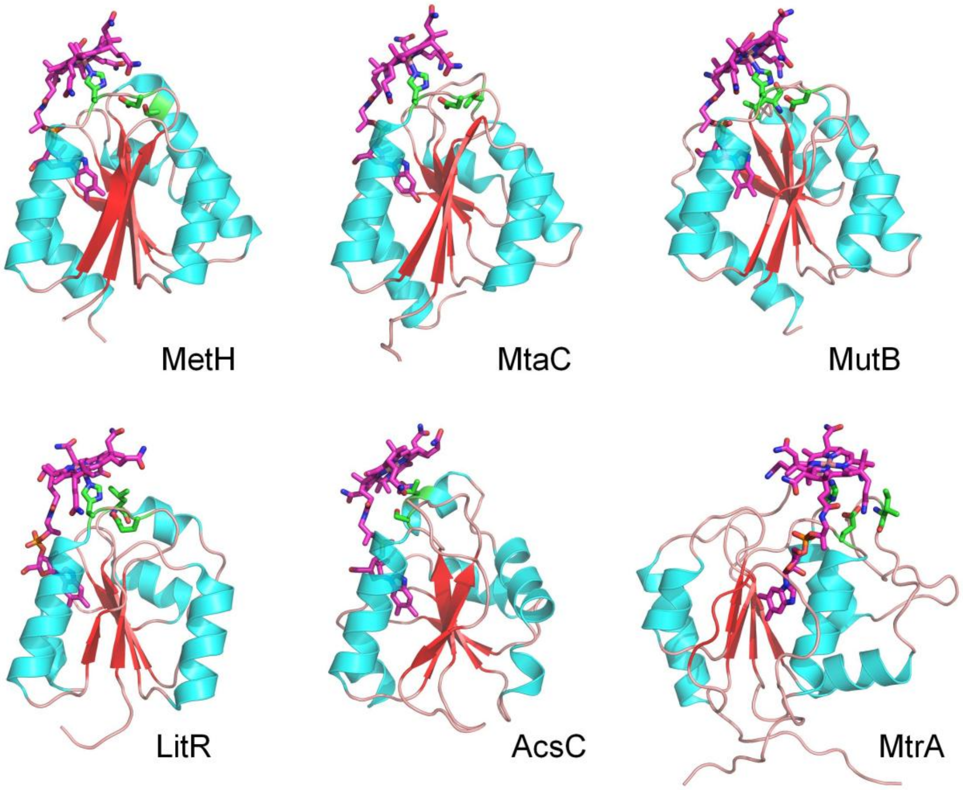

Supplementary Figure S11. Protein fold and position of cobalamin binding in Rossmann-type B_{12}-binding proteins. MetH from *E. coli* (PDB: 1BMT), MtaC subunit (methanol:cobalamin methyltransferase complex) from *Methanosarcina barkeri* (PDB: 2I2X), MutB subunit (methylmalonyl-CoA mutase) from *Propionibacterium freudenreichii* subsp. *shermanii* (PDB: 1REQ), LitR (transcriptional regulator) from *Thermus thermophilus* (PDB: 3WHP), and AcsC subunit (acetyl-CoA synthase) of 5-methyltetrahydrofolate corrinoid/iron-sulfur protein methyltransferase from *Moorella thermoacetica* (PDB: 4DJD) and the MtrA homolog from *Methanothermus fervidus*. Stick models of cobalamin (carbons in pink) and amino acids involved in the hydrogen-bonding network at the axial side of cobalamin are shown (carbons in green).

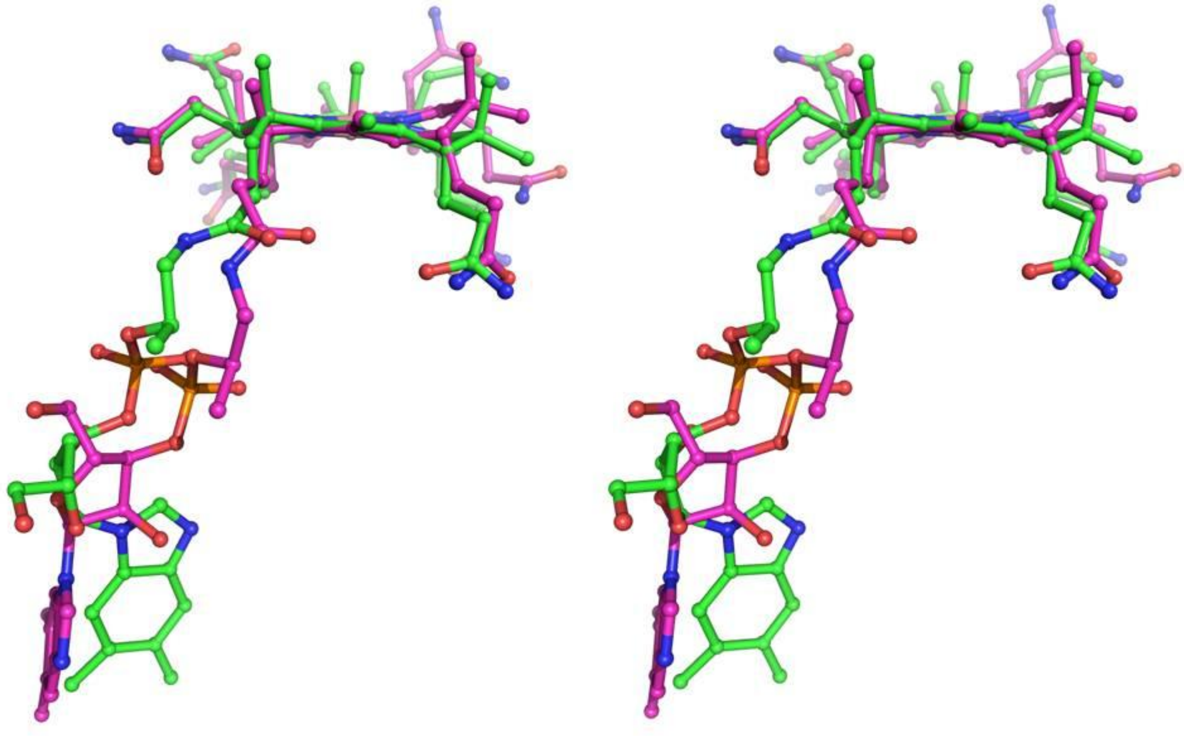

Supplementary Figure S12. Comparison of conformations of cobalamin bound to MtrA and MetH methyltransferases. Methyltransferase (MetH) from *E. coli* (green) and the cytoplasmic MtrA homolog from *Methanothermus fervidus* (pink) in stereo. An alignment of the cobalamin of MtrA and of methionine synthase MetH from *E. coli* which revealed a highly related conformation except for two propionamide side chains and the dimethylbenzimidazole group of MtrA; the latter is turned 90° in the direction of the cavity pocket (see Supplementary Fig. S8).

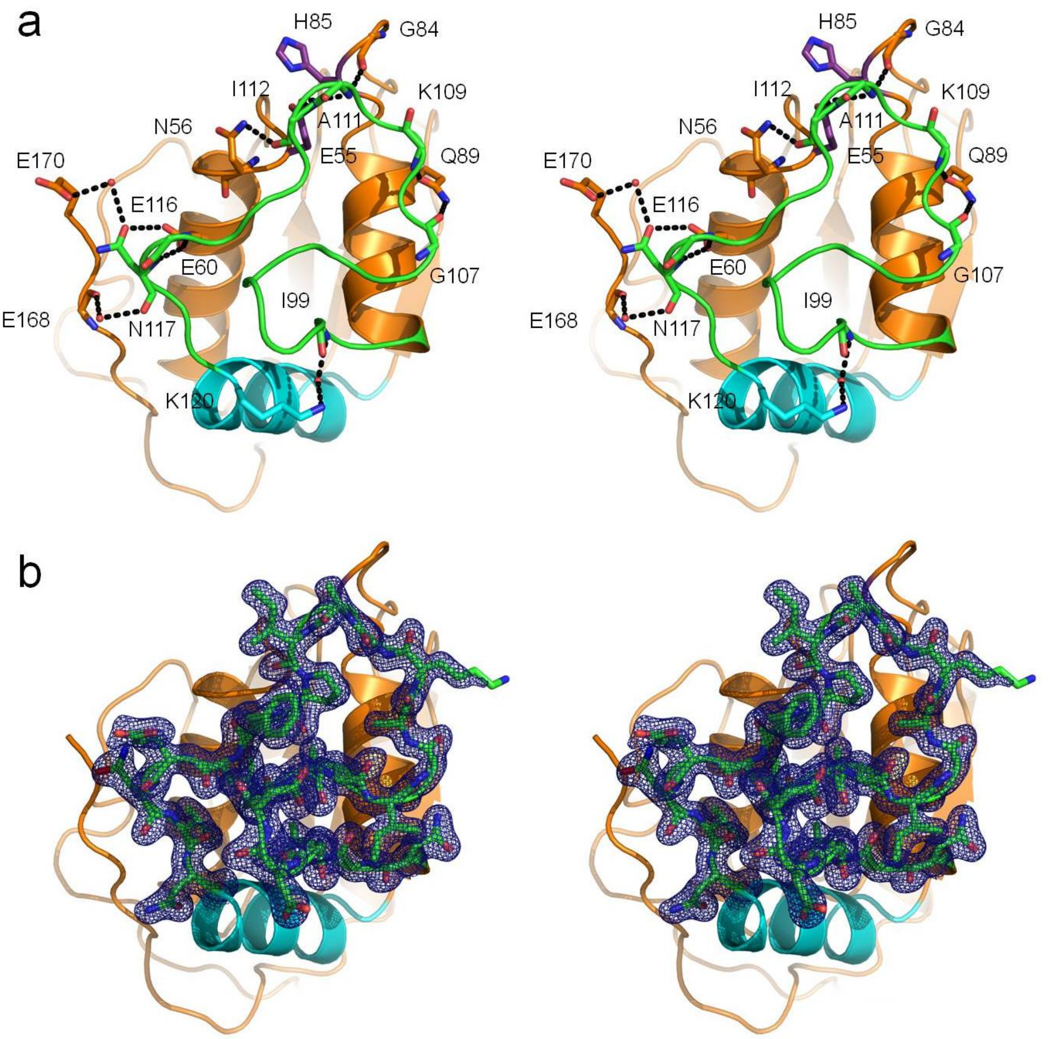

Supplementary Figure S13. The pronounced meander-like segment following helix α3 of the MtrA from *Methanocaldococcus jannaschii*. a) Cartoon with hydrogen-bonding between the meander-like segment and the core region. **b**) The $2F_o$-F_c map at the meander-like segment is contoured at 1.0 σ (blue mesh).

Supplementary Table 1 | Data collection and refinement statistics.

	The cytoplasmic MtrA homolog *Methanothermus fervidus* (SAD[a])	The soluble domain of membrane-associate MtrA from *Methanocaldococcus jannaschii*
**Data collection**		
Wavelength (Å)	1.602	1.000
Space group	*I*422	$P2_1$
Resolution (Å)	93.95–3.00 (3.18–3.00)	45.11–1.85 (1.95–1.85)
Cell dimensions		
a, b, c (Å)	100.6, 100.6, 262.7	64.69, 37.03, 65.16
α, β, γ (°)	90.00, 90.00, 90.00	90.00, 91.98, 90.00
R_{merge} (%)[b]	22.4 (105.7)	12.3 (60.0)
R_{pim} (%)[b]	7.4 (44.4)	9.3 (45.1)
I/σ_I[b]	8.6 (2.5)	6.1 (2.1)
Completeness (%)[b]	98.5 (91.0)	95.4 (95.6)
Redundancy[b]	17.1 (11.9)	2.3 (2.3)
**Refinement**		
Resolution (Å)	93.95–3.00	45.11–1.85
Number of reflections	13073	25433
R_{work}/R_{free}[c] (%)	21.1/25.4	18.9/23.7
Number of atoms		
Protein	3,644	2,569
Ligands/ions	273	18
Solvent	0	101
Average B-factors ($Å^2$)	49.9	24.6
MolProbity clash score, all atoms	3.3 (100th percentile)	2.15 (99th percentile)
Ramachandran plot		
Favoured regions (%)	452 (95.36)	331 (97.35)
Outlier regions (%)	0 (0)	1 (0.29)
rmsd[d] bond lengths (Å)	0.01	0.009
rmsd[d] bond angles (°)	2.302	1.197

[a] Single anomalous dispersion

[b] Values for the highest resolution shell are written within parentheses.

[c] R_{free} was calculated for 5% of the reflections that were not included in the refinement.

[d] rmsd, root mean square deviation.
